# Case Report: Ovulation Induction in Greater One-Horned Rhinoceros (*Rhinoceros unicornis*)

**DOI:** 10.3389/fvets.2021.657284

**Published:** 2021-06-30

**Authors:** Robert Hermes, Folko Balfanz, Simone Haderthauer, Eveline Dungl, Thomas B. Hildebrandt, Franz Schwarzenberger

**Affiliations:** ^1^Leibniz Institute for Zoo and Wildlife Research, Berlin, Germany; ^2^Tierärztliche Ordination Tiergarten Schönbrunn, Vienna Zoo, Vienna, Austria; ^3^Vienna Zoo, Vienna, Austria; ^4^Department for Biomedical Sciences, University of Veterinary Medicine, Vienna, Austria

**Keywords:** Indian rhinoceros, hCG, GnRH analog, ultrasound, endocrinology, estrus

## Abstract

Despite a profound knowledge on reproduction biology in greater one-horned (GOH) rhinoceros, many individuals cope with sub or infertility or an-ovulatory estrous. At the same time, early and regular captive breeding is of high importance in female GOH rhinoceros due to their high prevalence to develop genital tract tumors with consequent cessation of reproduction. Thus, mature, an-ovulatory GOH rhinoceros represent a challenge for captive breeding programs and warrant for means of reliable ovulation induction. Here, we used hCG and GnRH analog histrelin to induce ovulation in an-ovulatory GOH rhinoceros. Upon ultrasound diagnosis of a preovulatory follicle hCG or GnRH were injected to induce ovulation (*n* = 11). As a result, 75% of the hCG (*n* = 6/8) and 33% of GnRH (*n* = 1/3) inductions resulted in ovulation. Ovulation occurred when fecal estrogen concentration increased before and pregnane concentration after induction. Thirty-six percent of all treatments (*n* = 4/11) failed to induce ovulation. When ovulation induction by hCG/GnRH injection failed, estrogen and pregnane concentrations were significantly lower compared to ovulatory estrous (*P* < 0.001). Our results suggest that hCG and GnRH analog facilitate an easily applicable treatment to induce ovulation in females with behavioral but at times an-ovulatory estrous. Frequent use of hCG as an ovulation inducer might help to achieve pregnancies in genetically important but an-ovulatory GOH rhinoceroses.

## Introduction

The mean estrous cycle length of the greater one-horned (GOH) rhinoceros is 43 ± 6 d (1, 2). Like all other rhinoceros species, the GOH rhinoceros (*Rhinoceros unicornis*) is mono-ovulatory. Follicles grow in waves, from which the dominant, preovulatory follicle develops. The preovulatory, Graafian follicle measures an exceptional 10–12 cm in diameter, which is much larger than in other rhinoceros species. In fact, with a volume of 300–500 mL, it is the largest ovulatory follicle described for any mammal species (2).

Despite available profound knowledge on reproduction biology in GOH rhinoceros, many individuals are affected by sub- or infertility, silent estrous, anestrous, early embryonic death, or still birth (3–5). Early and regular captive breeding is of high importance in female GOH rhinoceros due to their high prevalence to develop genital tract tumors and consequent cease of reproduction by the age of 18 years in captivity (4). So far, assisted reproduction using fresh and frozen-thawed semen in artificial inseminations (AIs) has been used (5). Pregnancy rates after AI of up to 50% were achieved upon ovulatory estrous cycles (5).

Estrous synchronization in anestrous females to achieve ovulation and conception after natural mating or AI is an assisted reproduction tool so far described in black and white rhinoceros (6–8). For this, synthetic progestin, altrenogest, and chlormadinone acetate, combined with a hCG or a GnRH analog has been used to synchronize estrous and trigger ovulation (8–10). Oral progestin ceases folliculogenesis for the duration of the treatment. Removal of the oral progestin induces a rebound effect on the hypothalamus-pituitary axis, which initiates the growth of a new follicular wave and development of an ovulatory follicle. In anestrous white rhinoceros, oral progestin treatment paired with GnRH analog deslorelin implants resulted in increased luteal activity suggesting an ovulation rate of 93.3% (4). Consequently, use of oral estrous synchronization protocols have led to multiple pregnancies after natural mating and assisted reproduction in anestrous white and black rhinoceros (8, 9, Hermes, unpubl data). In addition, ovulation induction using GnRH analogs has also been described in white rhinoceros (11, 12).

In GOH rhinoceros, irregular, and an-ovulatory estrous cycles with subsequent formation of hemorrhagic follicles represent a challenging problem in captive breeding programs. However, estrous synchronization has yet not been described for GOH rhinoceros. Only ovulation induction has been briefly mentioned being used during AIs (2, 5). Here, exogenous GnRH analogs have been used to induce ovulation in two females with behavioral but sometimes an-ovulatory estrous. This preliminary data show that GnRH analogs, cystorelin, and deslorelin, induced five ovulations in a total of seven estrous cycles (2, 5). Yet, conclusions on the efficacy of GnRH analogs in inducing ovulation in GOH rhinoceros is limited, because the number of treatments is divided between different GnRH analog formulations and because treated females with behavioral estrous might have ovulated spontaneously even without administration of GnRH.

While hormone activity and ovarian dynamics can be monitored closely (1, 2), there are no reports on estrous synchronization, and there is limited data on ovulation induction in GOH rhinoceros. Therefore, we intended to test hCG and GnRH analog histrelin for their ability to induce ovulation in an-ovulatory GOH rhinoceros.

## Methods and Materials

### Study Animal and Hormone Monitoring

Ovulation induction was performed in one anestrous, 12-year-old, captive female GOH rhinoceros (studbook no: 367) over a study period of 4 years. The study animal is a wild born, orphan transferred from Nepal to the Vienna Zoo at the age of 3 years. The female is housed with a fertile male in separate enclosures. Continuous hormone analysis had been conducted since this female was 4 years of age. From the age of 9 years, regular peaks of estrogen-precursors indicated follicular development, but no clear pregnane peaks were measured, suggesting that ovulation never occurred.

Fecal samples were collected two times a week and analyzed using enzyme immunoassays for 20α-OH-pregnanes and 17-oxo-androstanes (1). Like estrogens, their precursors, the fecal 17-oxo-androstanes, are indicators of the follicular phase. Because fecal 17-oxo-androstanes have higher concentrations than estrogens, these estrogen precursors are routinely analyzed for reproductive monitoring of GOH rhinoceroses in the European Breeding Program. For monitoring hormone activity before and after ovulation induction, samples were analyzed at 2-day intervals.

### Transrectal Ultrasound and Treatment

The female has been trained to tolerate transrectal ultrasound examination (GE LOGIQ e Vet, GE Medical Systems, Wuxi 214028, China). Transrectal ultrasound monitored ovarian dynamics and development of preovulatory follicles. Weekly examinations were gradually increased toward daily ultrasounds when a dominant follicle emerged and grew >8 cm. Yet, without treatment pre-ovulatory follicles became atretic or hemorrhagic as previously described by Stoops et al. (2). The treatments to induce ovulation started when the animal was 12 years, at an age a regular estrous cycle should have long been established. When the preovulatory follicle (≥11 cm) was present for 48 h 10.000 IU hCG (Chorlulon 5000 I.U., MSD Tiergesundheit, A-1220 Vienna, Austria) or 1.5 mg of histrelin (Histrelin acetate biorelease, 0.5 mg/ml, Bet Pharm, Lexington, KY 40511, USA) were administered by intramuscular injection below the ear. In the first treatment series hCG was administered four times. This was followed by one time GnRH, two times hCG, two times GnRH, and finally two more times hCG. The interval between treatments ranged between 43 and 364 d. Irregular intervals between treatments were due to: (1) seasonal limitations to breed the female after ovulation induction during the winter season; (2) presence of persistent haemorrhagic follicles before new follicular waves started; and (3) periods of follicular inactivity during which no preovulatory follicles develop. Long intervals between treatments >43 d assured that possible drug-drug interactions could not occur when different hormones were used.

Success of the ovulation induction was determined by rupture of the Graafian follicle and the formation of a corpus luteum >48 h after hCG/GnRH injection and confirmed by endocrine monitoring.

### Statistics

For statistical analysis, day 0 represented the day of hCG injection. Hormone results were summarized in 3-day intervals starting −9 days before until +30 days after ovulation induction. Endocrine results of the treatments were grouped into those where pregnane increased at day 6 >100 ng/g feces and a corpus luteum had been diagnosed by ultrasound vs. those where pregnane at day 6 remained <100 ng/g feces and no corpus luteum was detected. Hormone results were compared using the Mann-Whitney Test and considered significantly different when *P* < 0.05.

## Results

**Figure 1:**
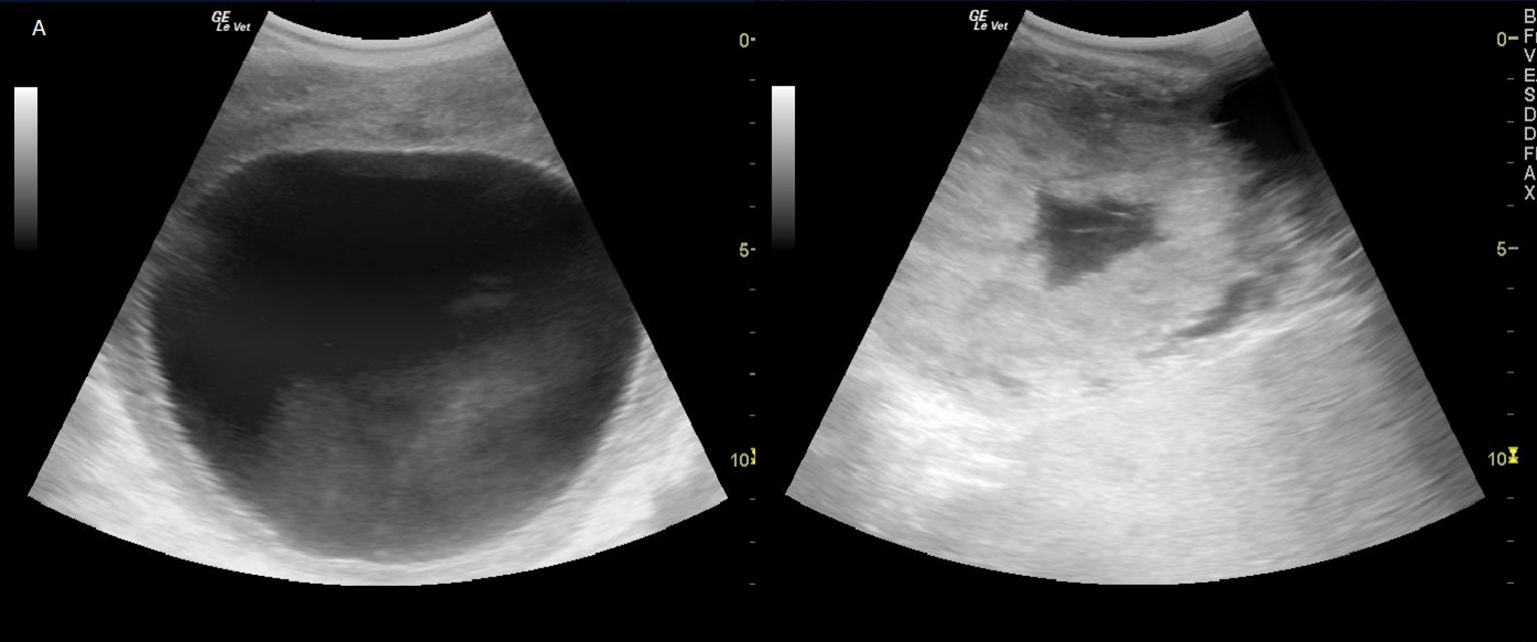
Left: Preovulatory follicle of ~11 cm in a greater one-horned rhinoceros. Right: Ruptured follicle with blood filled center shortly 48 h after ovulation induction.

The development of a preovulatory follicle in the an-ovulatory female took 26.9 ± 4.4 days (*n* = 9). A preovulatory follicle with a mean diameter of 11.9 ± 0.2 cm was imaged during eight silent estrous (Figure 1). When the preovulatory follicle had been present on the ovary for 48 h, ovulation was induced by i.m. injection of hCG or GnRH below the ear.

**Figure 2:**
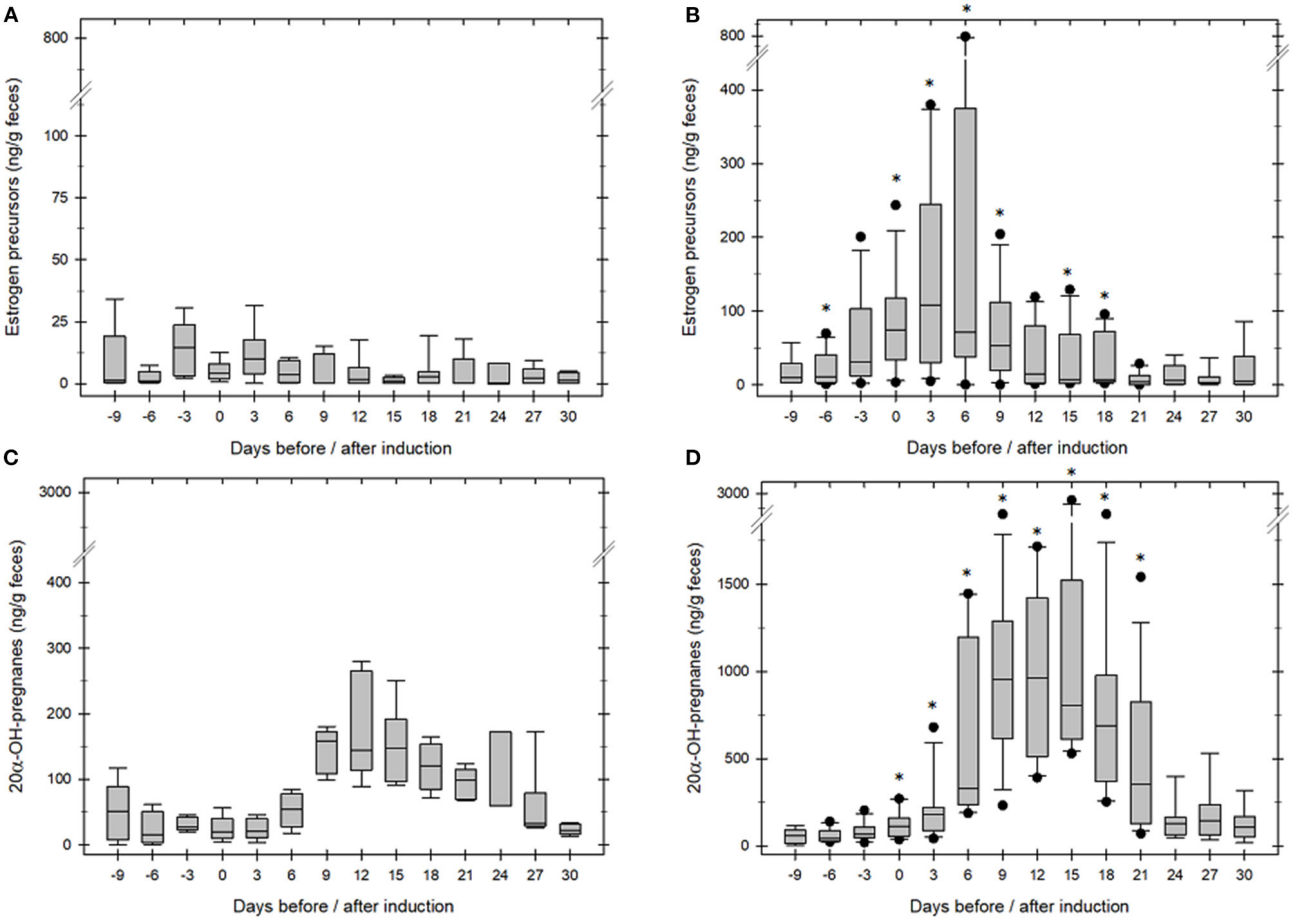
Fecal estrogen **(A,B)** and pregnane **(C,D)** concentration in anestrous GOH rhinoceros. Day 0 marks the day of hCG/GnRH injection. **(A,C)**: When ovulation was not induced median estrogen and pregnane remained low before and after treatment (*n* = 4). **(B,D)**: When ovulation was induced median estrogen increased significantly before (*p* ≤ 0.02) and gestagen significantly after ovulation (*n* = 7, *p* ≤ 0.01) compared to concentrations during an-ovulatory estrous **(A,C)**. *asterisk mark days median estrogen or pregnane concentrations were significantly different between groups (*p* ≤ 0.01).

Successful ovulation inductions (*n* = 7/11) were preceded by a significant, preovulatory increase of fecal estrogene concentration to >75 ng/ g feces. The presence of a 11.3 ± 0.9 cm corpus luteum (Figure 1) combined with a significant, postovulatory increase of 20α-OH-pregnanes concentration >750 ng/g feces confirmed ovulation and determined the success of the treatment (*P* < 0.005; Table 1, Figure 2). Four ovulation inductions (2 hcG/2 GnRH) failed to induce ovulation despite the presence of a preovulatory follicle. Inductions that failed were characterized by significantly lower, preovulatory estrogene concentrations <20 ng/g feces, absent formation of a corpus luteum and significantly lower pregnane concentrations from day 6 onward (Table 1; *P* < 0.005).

**Table 1:** Two-tailed *P*-values of the comparisons between high and low estrogen and pregnane concentrations during ovulation induction (*n* = 11) in one GOH rhinoceros.

**Fecal metabolite**	**−9 d**	**−6 d**	**−3 d**	**0 d**	**3 d**	**6 d**	**9 d**	**12 d**
Estogen	>0.05	=0.02	>0.05	**=0.002**	**=0.003**	**=0.01**	**=0.04**	>0.05
Pregnane	>0.05	>0.05	>0.05	**=0.002**	** <0.001**	** <0.001**	** <0.001**	** <0.001**

*Day 0 marks the day of induction. Significant difference in estrogen and pregnane on 0–9 d (bold) suggests that ovulation had been induced*.

HCG administered in the presence of a preovulatory follicle induced six ovulations resulting in an ovulation rate of 75% (*n* = 6/8). Injectable GnRH analog triggered ovulation only once (*n* = 1/3). Despite the rise in estrogen, the formation of a corpus luteum, and the increase in luteal activity as indicators for the induced ovulation, behavioral estrous was not observed before or after any of the hCG/GnRH treatments.

## Discussion

Anestrous and an-ovulatory rhinoceroses represent a challenge for captive breeding programs (1, 2, 5, 12–14). High genetic value of a female diagnosed with ovulation failure after natural mating or assisted reproduction requires means of reliable ovulation induction. Here we showed that injectable hCG or GnRH administered at the presence of a preovulatory follicle induced ovulation in one an-ovulatory GOH rhinoceros. Over both treatments ovulation induction was successful in 63.6% of the time.

So far, GnRH has been used incidentally for ovulation induction in two GOH rhinoceros (2, 5). GnRH was thought to help time ovulation for AI in females with behavioral sometimes an-ovulatory estrous (5). Mixed results using different GnRH analogs suggested that GnRH might not be reliable to induce ovulation in an-ovulatory GOH rhinoceros (2, 5). In this study, hCG repeatedly induced ovulation in a persistent an-ovulatory female GOH with silent behavioral estrous. In the presence of a preovulatory follicle, the administration of 10.000 IU hCG induced ovulation in 75% of the treatments. Despite the repeated use of hCG in the same animal, ovulation rate in the GOH rhinoceros was similar to ovulation rate of 78–87% reported in mares after single hCG injection (15). Thus, even when treated with hCG only once, mares did not show much higher ovulation rate compared to the repeatedly treated female GOH rhinoceros in this study. Injectable GnRH analog histrelin induced ovulation only once. Our data supports the broader use of hCG for ovulation induction in the presence of a preovulatory follicle in GOH rhinoceros. The combined number of previously reported GnRH inductions (2, 5) and those performed in this study (total *n* = 10) indicate that GnRH analogs are similarly suitable to induce ovulation in an-ovulatory GOH rhinoceros than hCG.

Results on the use of hCG for ovulation induction in GOH rhinoceros are consistent with results from white rhinoceros where 67% of estrous synchronizations using hCG resulted in ovulation (10). In white rhinoceros, the GnRH analog deslorelin has been superior to induce ovulation over hCG (10). Yet, the application of slow-release deslorelin implants is more challenging than i.m. injections and was therefore not attempted in this study (10).

On one hand, hormone-induced ovulation is technically challenging for many facilities and does not appear as a comprehensive method for reproduction management of captive GOH rhinoceros. It is likely limited to facilities with an advanced animal training program and where critical hardware such as an ultrasound machine and a restraint chute is present. On the other hand, in females at advanced age, with “erratic” an-ovulatory estrous cycles and conception failure but yet important genetics for the captive population, the use of hCG/GnRH during behavioral estrous might present a new, practical reproductive management tool.

In conclusion, our data shows that in presence of a preovulatory follicle hCG-induced ovulation in an-ovulatory GOH rhinoceros in 75% of treatments. Thus, injectable hCG represents an easy-to-apply and practical treatment to induce ovulation in GOH rhinoceros with behavioral but at times an-ovulatory estrous. As an-ovulatory GOH rhinoceros represents a challenge for captive breeding programs, frequent use of ovulation inducers, such as hCG or GnRH, might help to achieve late pregnancies in genetically important but an-ovulatory GOH rhinoceros.

## Data Availability Statement

The original contributions presented in the study are included in the article/supplementary material, further inquiries can be directed to the corresponding author/s.

## Ethics Statement

The animal study was reviewed and approved by Ethics Committee of the Leibniz Institute for Zoo and Wildlife Reserach. Written informed consent was obtained from the owners for the participation of their animals in this study.

## Author Contributions

RH: study design, data anaylsis, and manuscript writing and edit. FB: study design, data aquisitition, data analysis, and mansuscirpt edit. SH: Data auqiuisition. ED: data acquisition. TH: supervision and manuscript edit. FS: study design, data aquisition, data analysis, and manusciprt writing and edit. All authors contributed to the article and approved the submitted version.

## Conflict of Interest

The authors declare that the research was conducted in the absence of any commercial or financial relationships that could be construed as a potential conflict of interest.
